# Supplementary material for: FG nucleoporins feature unique patterns that distinguish them from other IDPs
Source: Biophys J. 2021 Jul 6;120(16):3382–91. doi: 10.1016/j.bpj.2021.06.031 (PMC8391076; doi:10.1016/j.bpj.2021.06.031)
Supplement: Document S1. Figs. S1–S3 [file mmc1.pdf]

Biophysical Journal, Volume *120*

Supplemental information

FG nucleoporins feature unique patterns that distinguish them from other IDPs

Mohaddeseh Peyro, Mohammad Soheilypour, Vikrum S. Nibber, Andrew M. Dickson, and Mohammad R.K. Mofrad

SUPPLEMENTARY MATERIALS

FG Nucleoporins feature unique amino acid sequence patterns that distinguish them from other IDPs

Mohaddeseh Peyro[1,§], Mohammad Soheilypour[1,§], Vikrum S. Nibber[1], Andrew M. Dickson[1], Mohammad R.K. Mofrad[1,2,*]

[1] Molecular Cell Biomechanics Laboratory, Departments of Bioengineering and Mechanical Engineering, University of California, Berkeley, Berkeley, CA 94720
[2] Molecular Biophysics and Integrative Bioimaging Division, Lawrence Berkeley National Laboratory, Berkeley, CA 94720

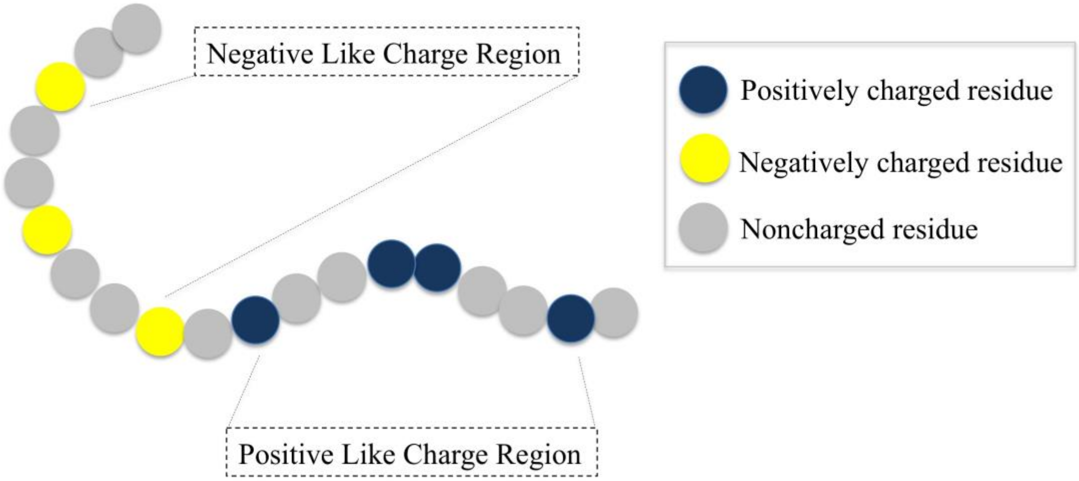

Fig. S 1: Hypothetical disordeded region of a protein containing a positive like charge region (LCR), with a length of 8 and charge count of 4, followed by a negative LCR, with a length of 7 and charge count of 3.

§These authors contributed equally to this work.
*Corresponding author: mofrad@berkeley.edu

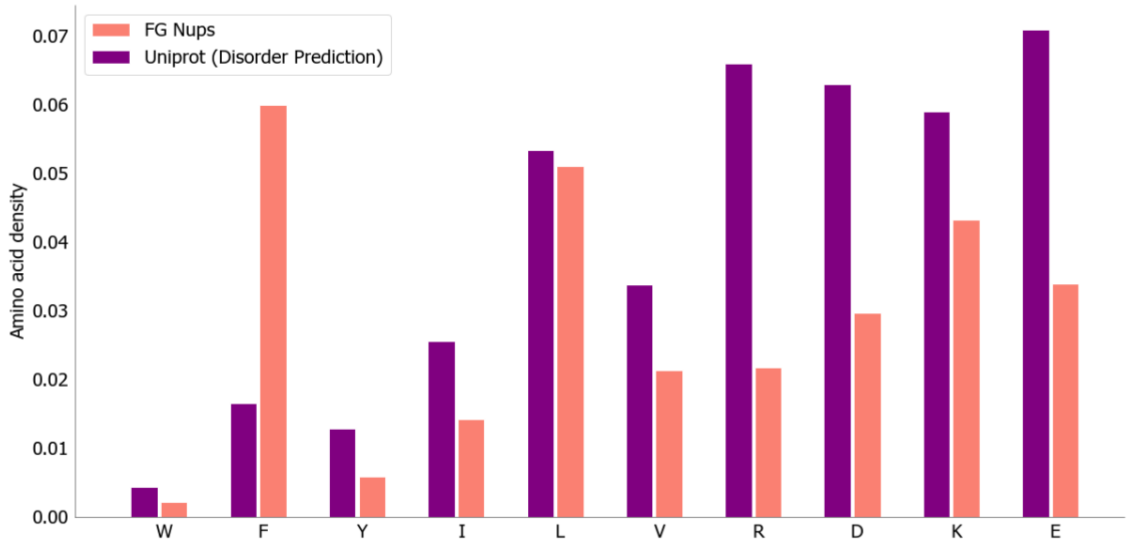

Fig. S 2: Comparison of abundance of the most order-promoting and the most disorder-promoting amino acids in FG Nups vs. UniProt (disorder prediction). FG Nups have a lower abundance of all of the order-promoting amino acids except for F. The sum of the abundance of order-promoting amino acids are nearly equal. On the other hand, the abundance of all of the charged residues is lower in FG Nups compared to UniProt (disorder prediction)

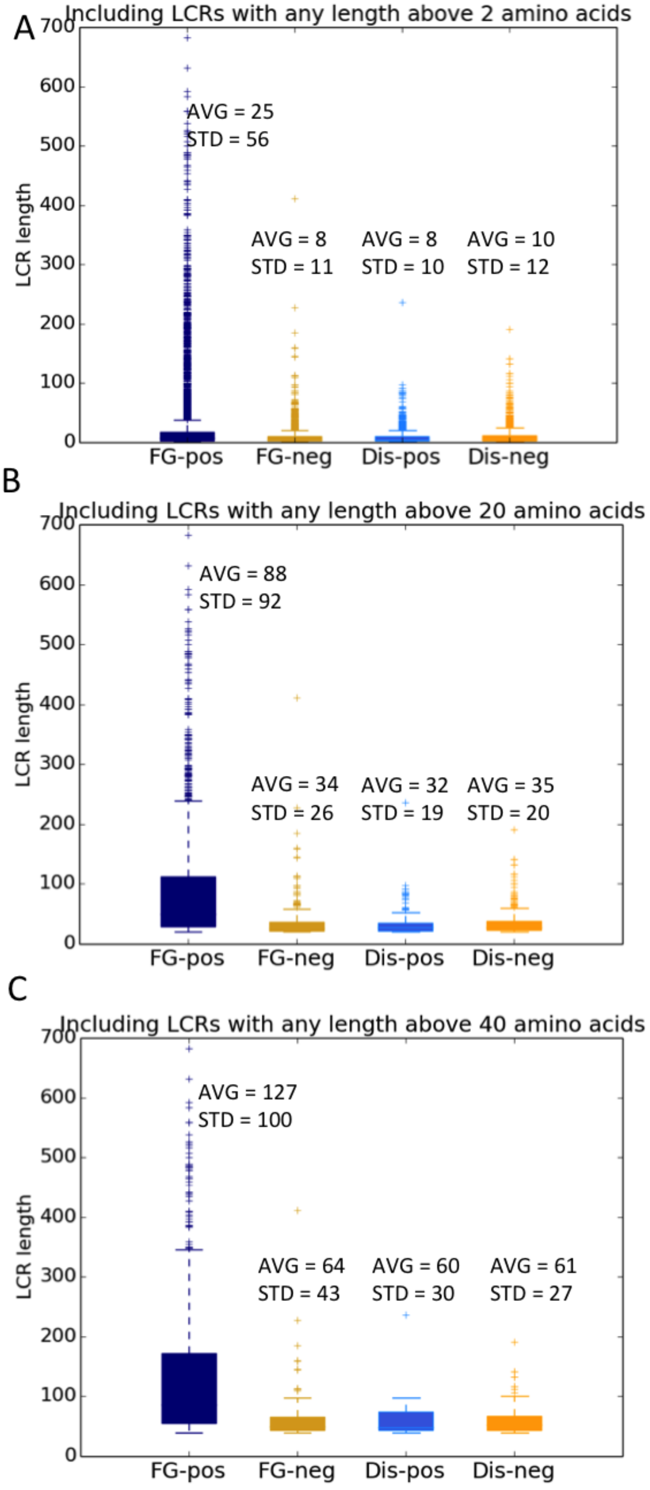

Fig. S 3: Boxplots of length of positive LCRs in FG Nups, negative LCRs in FG Nups, positive LCRs in DisProt and negative LCRs in DisProt A) Considering LCRs of any length (above 2 amino acids), the average length of positive LCRs in FG Nups is 3 times higher than the other three categories. Panels (B) and (C)) Removing shorter LCRs (below 20 in panel B and below 40 in panel C) increases the average length of all the categories, but average length of positive LCRs in FG Nups remains significantly higher than the other three categories, and emphasizes the difference in the length distribution of positive LCRs in FG Nups.
