# Supplementary material for: FG nucleoporins feature unique patterns that distinguish them from other IDPs
Source: Biophys J. 2021 Jul 6;120(16):3382–91. doi: 10.1016/j.bpj.2021.06.031 (PMC8391076; doi:10.1016/j.bpj.2021.06.031)
Supplement: Document S2. Article plus supporting material [file mmc2.pdf]

Biophysical Journal
Article

Biophysical Society

FG nucleoporins feature unique patterns that distinguish them from other IDPs

Mohaddeseh Peyro,[1] Mohammad Soheilpour,[1] Vikrum S. Nibber,[1] Andrew M. Dickson,[1] and Mohammad R. K. Mofrad[1,2,*]

[1]Molecular Cell Biomechanics Laboratory, Departments of Bioengineering and Mechanical Engineering, University of California Berkeley and [2]Molecular Biophysics and Integrative Bioimaging Division, Lawrence Berkeley National Laboratory, Berkeley, California

ABSTRACT FG nucleoporins (FG Nups) are intrinsically disordered proteins and are the putative regulators of nucleocytoplasmic transport. They allow fast, yet selective, transport of molecules through the nuclear pore complex, but the underlying mechanism of nucleocytoplasmic transport is not yet fully discovered. As a result, FG Nups have been the subject of extensive research in the past two decades. Although most studies have been focused on analyzing the conformation and function of FG Nups from a biophysical standpoint, some recent studies have investigated the sequence-function relationship of FG Nups, with a few investigating amino acid sequences of a large number of FG Nups to understand common characteristics that might enable their function. Previously, we identified an evolutionarily conserved feature in FG Nup sequences, which are extended subsequences with low charge density, containing only positive charges, and located toward the N-terminus of FG Nups. We named these patterns longest positive like charge regions (lpLCRs). These patterns are specific to positively charged residues, and negatively charged residues do not demonstrate such a pattern. In this study, we compare FG Nups with other disordered proteins obtained from the DisProt and UniProt database in terms of presence of lpLCRs. Our results show that the lpLCRs are virtually exclusive to FG Nups and are not observed in other disordered proteins. Also, lpLCRs are what differentiate FG Nups from DisProt proteins in terms of charge distribution, meaning that excluding lpLCRs from the sequences of FG Nups make them similar to DisProt proteins in terms of charge distribution. We also previously showed the biophysical effect of lpLCRs in conformation of FG Nups. The results of this study are in line with our previous findings and imply that lpLCRs are virtually exclusive and functionally significant characteristics of FG Nups and nucleocytoplasmic transport.

SIGNIFICANCE The nuclear pore complex (NPC) and nucleocytoplasmic transport (NCT) have attracted significant attention because they are correlated with many pathological conditions such as cancer, autoimmune diseases, or genetic disorders. Despite numerous studies, the complex molecular machinery behind NCT function is not fully understood. Understanding the underlying mechanism of NPC function is the first step toward uncovering these pathological conditions. The function of NPCs mainly relies on FG nucleoporins (FG Nups) and the regulatory role they play in NCT. This study suggests that FG Nups contain specific evolutionarily conserved amino acid sequence features that are not observed in other disordered proteins and hence might be functionally significant for the role of FG Nups in NCT.

INTRODUCTION

The nuclear pore complex (NPC) is the largest macromolecule in the cell and is responsible for bidirectional transport of cargo through the nuclear envelope. Nucleocytoplasmic transport is vital for the cell and is mainly regulated by intrinsically disordered proteins named FG nucleoporins (FG Nups). These proteins are named FG Nups because they are rich in phenylalanine-glycine repeats. FG Nups enable fast, yet selective, transport through the NPC. The transport process through the NPC has been the focus of a large body of research for the past few decades. These studies have investigated this protein complex from different perspectives, including, but not limited to, the structure of the NPC (1,2) and transport selectivity (3–5). In recent years, however, researchers have found that to better understand the transport process and the specific role of FG Nups in the transport process, they need to pay closer

Submitted August 5, 2020, and accepted for publication June 16, 2021.

*Correspondence: mofrad@berkeley.edu

Mohaddeseh Peyro and Mohammad Soheilpour contributed equally to this work.

Editor: Rohit Pappu.

https://doi.org/10.1016/j.bpj.2021.06.031

© 2021 Biophysical Society.

This is an open access article under the CC BY license (http://creativecommons.org/licenses/by/4.0/).

attention to the amino acid sequences of FG Nups (6–13). Similarly, the sequence-conformation-function relationships in unstructured proteins in general have been the subject of substantial research in the past decade (14–22).

Studying disordered proteins and their conformations has become increasingly popular with the growing number of intrinsically disordered proteins (IDPs) and IDP regions (IDPRs) discovered (23). IDPs and IDPRs are known to lack a fixed secondary structure because of their specific amino acid sequence characteristics (24–27). In the past decade, many studies have focused on studying the sequences of disordered proteins in detail and investigated how amino acid sequences correlate with conformational properties of IDPs. It has been shown that IDPs with significantly different amino acid sequences can feature specific "evolutionary signatures" in their amino acid sequence that rescue their function (28–30). It is also well established that the presence of several uncompensated charge groups causes IDPs or IDPRs to have a net charge. This feature and low hydrophobicity content are the two major factors that prevent IDPs and IDPRs from forming a fixed secondary structure (26,31,32). In addition, IDPs and IDPRs are known to have a high content of disorder-promoting amino acids (24). IDPs and IDPRs are shown to form two general conformations, namely intrinsic coil and intrinsic premolten globule. The intrinsic coil conformation has a more relaxed-coil shape, whereas the premolten globule shows a more compact conformation. Although the premolten globules are closer to structured proteins in terms of conformation, they still fall under the umbrella of IDPs (27).

Among the sequence properties that influence the conformation of IDPs, the effect of charged residues is more widely investigated (14–22). Fraction of positive and negative charges f_+ and f_-, respectively; net charge per residue ($f_+ - f_-$); and fraction of charged residues ($f_+ + f_-$) in the sequence were early metrics employed to explore this effect (15,18,27). The combination of these metrics can classify IDPs into different conformational classes (17,18). However, because these are averaged metrics, they can only explain general characteristics of IDPs and are not detailed enough to model the effect of charge decoration on the conformation of specific FG Nups subsequence. Another metric, κ, was introduced by Das and Pappu and can effectively capture the effect of distribution of opposite charges on global conformation of IDPs (17). In addition, more studies explored the correlation between charged patterning and conformation of IDPs. Sequences with the same charge content but different charge patterning were shown to have different conformations (17,33,34). For instance, Huihui et al. showed that mutation of even one charged residue can induce significant changes in conformation of IDPs (20). Therefore, recent studies focus more on correlating the charge decoration with conformation of IDPs. For example, Firman and Ghosh showed that charge decoration in IDP sequences determines whether they have a coil or globule conformation (35). Focusing on a specific type of proteins named polycomb group (PcG) proteins, Beh et al. observed absence of negative charges in long stretches of amino acid sequences of these proteins and showed that this specific charge decoration is correlated with function of PcG proteins (36).

Although the sequence composition, conformation, and function of FG Nups have been studied before (6,7,9–11,13,37), very few studies have investigated the effect of charge decoration on FG Nups, their conformation, and their function (6,12,13). In our previous study, we found that FG Nups contain specific sequence features named longest positive like charge regions (lpLCRs) that are evolutionarily conserved across more than 250 species (12). These patterns are extended subsequences that have a long length and a low charge density and that only feature positively charged amino acids (see Fig. 1). Of note, negatively charged amino acids did not show such a distinct pattern. As a follow-up to our previous study, here we explore whether these features are specific to FG Nups or could be found in other disordered proteins as well. Accordingly, we analyzed two large data sets of disordered proteins, i.e., DisProt and disorder prediction of UniProt, for the presence of lpLCRs. Our results suggest that lpLCRs are amino acid sequence patterns (evolutionary signatures) virtually exclusive to FG Nups and do not appear in other disordered proteins, here represented by DisProt and disorder prediction of UniProt, which further suggests that lpLCRs may be important exclusive FG Nup features that dictate their specific function in nucleocytoplasmic transport.

MATERIALS AND METHODS

Most analyses presented herein were conducted via in-house Python scripts, except disorder prediction, which was conducted using ESpritz (version 1.3). Data sets, and Python scripts are all available at https://github.com/molecular-cell-biomechanics-lab.

Protein data sets

Three groups of proteins were analyzed in this study; the first group, named FG Nups, was taken from our previous study (12). This data set includes

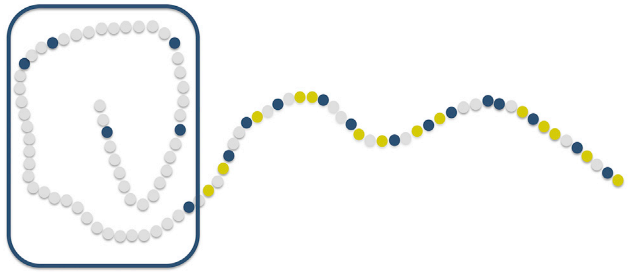

FIGURE 1 Schematic of the disordered domain of an FG Nup and its lpLCR. Blue residues are positively charged, and gold residues are negatively charged. To see this figure in color, go online.

1138 FG Nups from 252 species. This data set was originally extracted from UniProt by extracting entries associated with the keyword "nucleoporin" and choosing proteins with a high percentage of disorder (>30%) and high percentage of FG motifs (>0.15 FG/AA) (8). The disordered domains of these proteins were extracted and used in all the analysis, using the ESpritz-x prediction method (38). The second group, termed DisProt, included more than 1500 disordered regions of 694 proteins adapted from the DisProt database (39,40). DisProt is a database of proteins that contain at least one experimentally determined disordered domain along their sequence. The third group, named UniProt (disorder prediction), was downloaded from https://d2p2.pro, where ESpritz-x (38) prediction of all proteins was available (41).

Sequence analysis

Abundance of residues is defined as the number of target residues divided by the total length of the disordered region of the protein. An LCR is defined as a region that starts at the first position of a certain charge and continues until reaching an opposite charge in the amino acid sequence. The residues that were considered negatively charged were aspartic acid (D) and glutamic acid (E), and the positively charged residues included lysine (K) and arginine (R). For example, Fig. S1 shows a hypothetical positive LCR with a length of 8 and charge count of 4, followed by a negative LCR with a length of 7 and charge count of 3. LCR charge content is defined as the number of charged residues divided by the total length of the LCR. LCR-covered percentage is defined as the sum of length of LCRs in a protein sequence divided by the total length of its disordered region.

RESULTS

Charged residue abundance and charged residue distribution are different in FG Nups versus DisProt proteins

In this study, we analyzed the presence of negative and positive LCRs in disordered domains of FG Nup and DisProt proteins. LCRs are defined as any stretch of an amino acid sequence that contains only one type of charged amino acids: either positively charged (lysine, arginine) or negatively charged (aspartic acid, glutamic acid) (Fig. 1). In each data set, only the disordered domains were analyzed, and the method of extraction of each of these data sets is explained in the Materials and methods.

IDPs and IDPRs are known to be rich in charged residues and have a low density of hydrophobic residues compared to structured proteins. Therefore, we compared the abundance of these amino acids between the two data sets (Fig. 2 *A*).

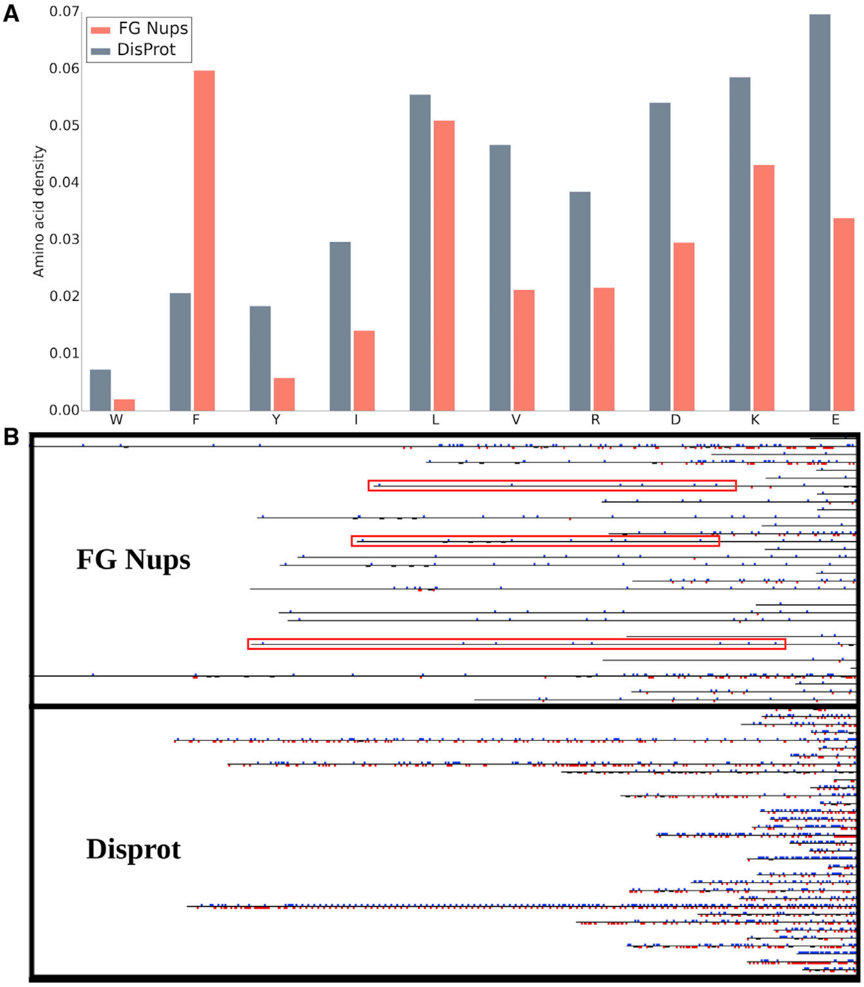

FIGURE 2 (*A*) Comparison of abundance of the most order-promoting and the most disorder-promoting amino acids in FG Nups versus DisProt. FG Nups have a lower abundance of all of the order-promoting amino acids except for F. The sums of the abundance of order-promoting amino acids are nearly equal. On the other hand, the abundance of all of the charged residues is lower in FG Nups compared with DisProt. The abundance of the analyzed amino acids is very similar in DisProt and disorder prediction of UniProt. Therefore, they compare similarly with FG Nups (please see Fig. S2). (*B*) A snapshot of a small part of the schematic comparing charged residue distribution in FG Nups versus DisProt (the entire schematic was too large to be presented). The figure shows that FG Nups have a lower charged residue density compared to DisProt and that DisProt has a lower average length compared to FG Nups. The most interesting difference is that in the FG Nup sequences, several regions can be observed that are extensively large segments of the protein that have a low charge density and only have positively charged residues. Some of these regions are boxed with red. We have named these regions longest positive like charge regions (lpLCRs). The proteins in the FG Nups data set depict this pattern very frequently. Many of the FG Nups have two segments in their sequence; one is charge rich (similar to sequences in DisProt), and the other has a low density of charged residues that are almost always positively charged (lpLCR, the *boxed pattern*). Some of the FG Nups also do not contain the charge-rich part and are only composed of an lpLCR. To see this figure in color, go online.

The amino acids are shown in the order of order-promoting to disorder-promoting property (42,43). FG Nup proteins have a lower abundance of all of the hydrophobic amino acids except for phenylalanine (Phe). A higher abundance of Phe is expected because of the naturally high abundance of Phe and Gly in FG Nups. The lower abundance of W, Y, I, L, and V in FG Nups is approximately compensated by the higher abundance of F, considering that F is the second most order-promoting residue. Therefore, the two data sets have an almost equal abundance of order-promoting amino acids. On the other hand, the abundance of all of the charged residues is higher in DisProt proteins compared with FG Nups. Charged residues are the most disorder-promoting types of amino acids. This means that although FG Nups have the same abundance of order-promoting amino acids as those in DisProt amino acids, they feature a lower density of disorder-promoting amino acids.

The same analysis was performed on the data set containing the disorder prediction of UniProt (please see Fig. S2). The abundance of amino acids is very similar in DisProt and the disorder prediction of UniProt. As a result, they compare similarly with FG Nups, showing a nearly equal abundance of order-promoting amino acids and a higher abundance of charged residues.

Subsequently, we compared charge distribution between the FG Nup and DisProt data sets. A schematic of charge distribution of all of the proteins in the two data sets was drawn. The complete data sets were too large to be presented here; therefore, a snapshot of part of the schematic for each of the data sets is presented (Fig. 2 *B*). Although this is a small fraction of the entire data set, it represents a meaningful comparison between the charge distribution in FG Nups and in DisProt. The black lines represent the amino acid sequences, the small vertical red lines depict negatively charged residues, and the small blue lines depict the positively charged residues. Only charged residues are represented in this schematic for the sake of clarity.

The first noticeable difference is that DisProt sequences are more "colorful," implying that DisProt proteins have a higher charged residue density, which is supported by Fig. 2 *A*, in which the abundance of charged residues is compared in the two data sets. The second difference is that on average, the length of protein sequences in the DisProt database is shorter than that in FG Nups. The third and main difference is that in the FG Nup sequences, several extensively large subsequences exist that have a low charge density and only contain positively charged residues (i.e., lpLCRs). Some of these regions are boxed with red. The proteins in the FG Nups data set depict this pattern very frequently. Additionally, many of the FG Nups have two segments in their sequence; one is charge rich (similar to sequences in the DisProt database), and the other has a low density of charged residues that are positively charged (lpLCR, the *boxed pattern*). Notably, some of the FG Nups do not contain the charge-rich segment and are only composed of an lpLCR.

Long subsequences that contain only a few positively charged residues named lpLCRs are virtually exclusive to FG Nups and do not exist in DisProt or disorder prediction of UniProt proteins

To gain a better insight into the characteristics of positively charged and negatively charged residues in these three data sets, the amino acid sequence length and charge contents in the longest LCR region (either positively or negatively charged) of each protein in each data set were extracted and compared between FG Nups, DisProt, and UniProt (disorder prediction) (Fig. 3; please see Materials and methods for definition of these data sets). Please note that disorder prediction of UniProt contains almost 500,000 proteins. For the comparison to be accurate, in the data used for this figure, the FG Nups are removed from the UniProt data set. LCR length is the number of amino acids between the charged residue that the LCR starts with and the charged residue that the LCR ends with (Fig. S1). LCR charge content (number density) is the number of charged residues present in the LCR divided by the number of amino acids in the LCR. A clear distinction can be observed between the three graphs in terms of the distribution of longest positive LCR data points. In the DisProt and UniProt (disorder prediction) data sets, the longest positive and negative LCR regions are almost entirely found on the left side of the graph. These longest LCRs can have widely varying charges, but fewer than 1 in 5000 (0.02%) exceed a length of around 200 residues. In the FG Nups data set, a similar pattern can be observed on the left side of the graph, on which the longest positive and negative LCRs with short length are present. However, the distinctive feature of the FG Nups data set is the presence of lpLCRs that are long but have a low charge density (boxed in *red*). Being a very large data set, UniProt (disorder prediction) has very few lpLCR data points of long length as well. Our analysis shows that there are almost 100 outlier proteins. 40% of these are FG Nup open reading frames (ORFs) with a high FG content. Out of the remaining, 20% are in the p21-activated kinase family or of miscellaneous category, whereas 40% are hypothetical or predicted proteins with no known function. This means that ~0.01% of the proteins in disorder prediction of UniProt are proteins that are not known FG Nups but feature lpLCRs larger than 200 amino acids, in contrast with the high fraction of lpLCRs in FG Nups. It is worth noting that the negative LCRs in the FG Nups data set do not show such an anomalous distribution and that this pattern is specific to positive LCRs. The average length of lpLCRs in FG Nups is 127 amino acids, whereas the average length of the longest negative LCRs is 19 amino acids. These values are 19 and 21 for the DisProt data set and 18 and 21 for UniProt (disorder prediction), respectively. Our results clearly demonstrate that the presence of long or low-charge-density lpLCRs is an almost exclusive characteristic of FG Nups.

As the next step, the positive and negative LCRs (of any length above two amino acids) of the FG Nups and DisProt

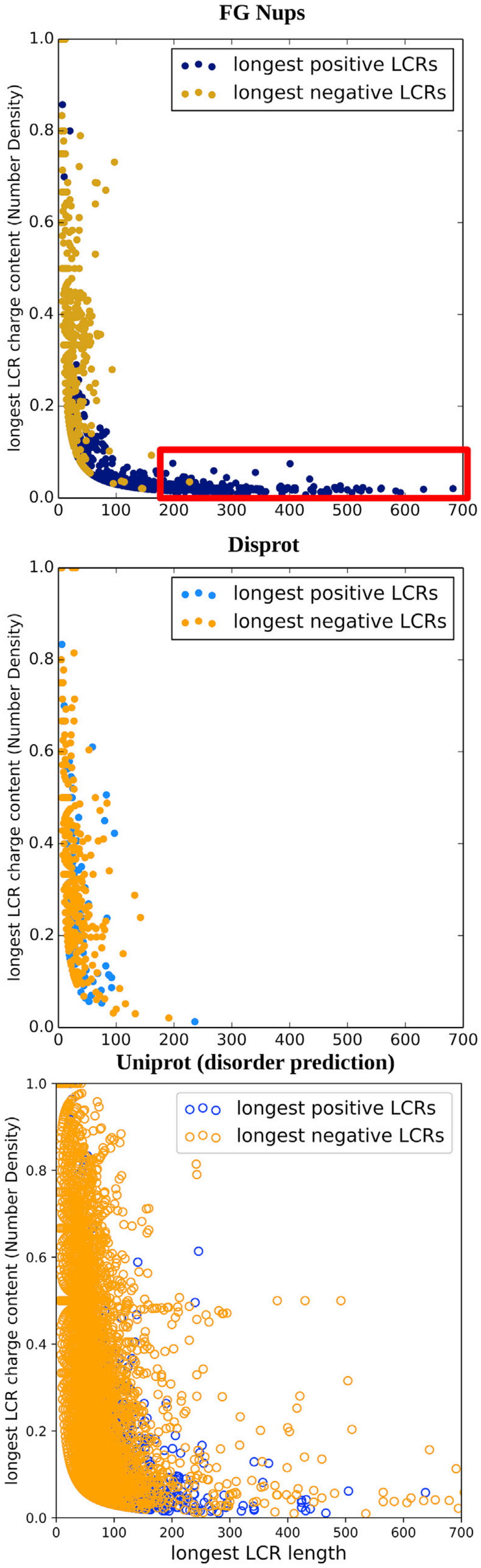

FIGURE 3 Scatterplots showing charge content plotted against LCR length for each data set. Three data sets (FG Nups, DisProt, and UniProt (disorder prediction)) are compared with each other in terms of their length and charge content of their LCRs. FG Nups are removed from disorder prediction of UniProt for a more accurate comparison. Please see the Materials and methods for definition of each data set. Only the longest positive and negative LCRs from each sequence were plotted, and only if the length was greater than two amino acids. The data show a clear distinction between FG Nups and the other two data sets. Only FG Nups feature lpLCRs that are long but have a low charge density (*boxed with red*). It is important to note that this pattern does not exist in longest negative LCRs in FG Nups, DisProt, or UniProt (disorder prediction). Therefore, these data suggest that the specific pattern observed in lpLCRs is specific to FG Nups and an extremely small fraction of other proteins. The outliers comprise around 100 proteins, 40% of which are FG Nup ORFs. Another 20% are in p21-activated kinase family or of miscellaneous category, and the other 40% are hypothetical or predicted proteins with no known function. These data suggest that almost 0.01% of proteins in disorder prediction of UniProt are proteins that are not known FG Nups but feature lpLCRs that are larger than 200 amino acids. However, this percentage is significantly low, and our results suggest that lpLCRs are virtually exclusive to FG Nups. To see this figure in color, go online.

data sets were compared (Fig. 4 *A*). It is important to note that these data are different from the data in Fig. 3 and include all of the LCRs present in the data set, not just the longest LCR in each protein. These results show that the majority of LCRs for all the four categories, i.e., positive LCRs in FG Nups, negative LCRs in FG Nups, positive LCRs in DisProt, and negative LCRs in DisProt, are short, charge-rich domains. However, the outliers of these data are different in these four groups. Positive LCRs in FG Nups have several outliers with extensively large length. These outliers cause the average length of positive LCRs in FG Nups to be significantly higher than the others (25 vs. 8 or 10). The very large standard deviation is also representative of the very large range that the data cover and reflects the presence of a significant number of outliers with large lengths.

Based on the results from Fig. 4 *A*, it was shown that a significant difference exists between the distribution of lengths of positive LCRs in FG Nups and the other three groups. Also, based on the observation from Fig. 3, FG Nups and DisProt are different in terms of the length and charge density of lpLCRs. We were interested to see whether the difference observed in the distribution of lengths of LCRs in Fig. 4 *A*, is caused only by presence of lpLCRs boxed in Fig. 3. Accordingly, we conducted the same comparison in Fig. 4 *A* while removing the lpLCR of FG Nups from the data (shown in Fig. 4 *B*). By removing the lpLCRs in FG Nups, the results look very similar across the four groups of data in terms of average, standard deviation, and outliers. As a result, it is reasonable to conclude that the major feature that differentiates FG Nup positive LCRs from other groups of data is the lpLCR in each sequence. Otherwise, the four groups of data look very similar. Collectively, our results demonstrate that lpLCRs in FG Nups, defined as long stretches of amino acids that have a low charged density and contain only positively charged residues, are virtually exclusive to FG Nups and the only region that

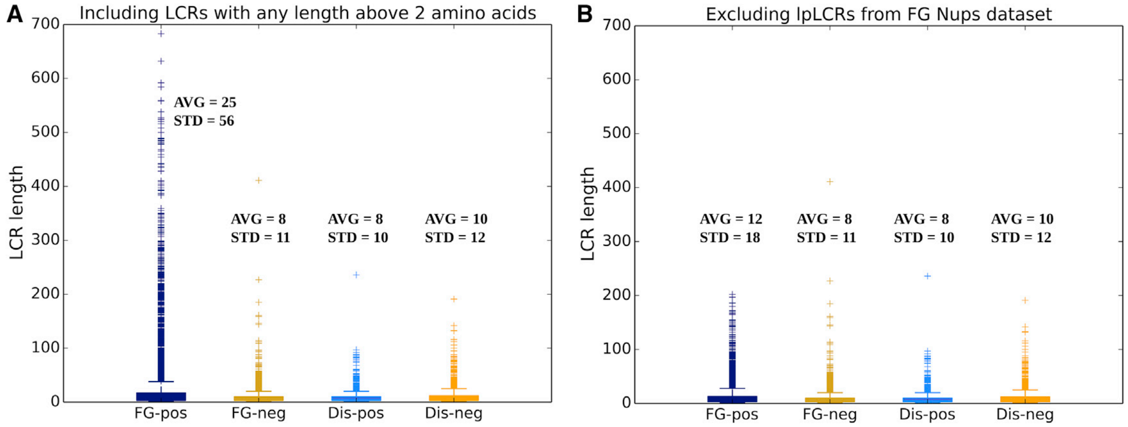

FIGURE 4 Boxplot of length of LCRs for positive LCRs in FG Nups, negative LCRs in FG Nups, positive LCRs in DisProt, and negative LCRs in DisProt. All of the LCRs above two amino acids in length are considered in these data sets. (*A*) Positive LCRs in FG Nups show a significant difference in terms of average and range from negative LCRs in FG Nups and positive and negative LCRs in DisProt. (*B*) lpLCRs in FG Nups are removed from the data, and the rest of the data look similar to the other three segments of data. This means that the major difference that can be observed in plot (*A*) between positive LCRs in FG Nups and the other three segments of data is due to presence of the lpLCRs in FG Nups, which mainly form the outlier section of positive LCRs. Other than that, the rest of the positive LCRs in FG Nups have similar characteristics to the other three segments of data. This implies that what differentiates FG Nups from DisProt in terms of charge distribution is the presence of lpLCRs. These patterns are virtually exclusive to FG Nups and are not observed in DisProt. AVG, average; STD, standard deviation. To see this figure in color, go online.

differentiates FG Nups from DisProt in terms of charge distribution.

Knowing that the lpLCRs in FG Nups are the major difference between the two data sets, we explored how defined and different the lpLCRs are compared with the other positive LCRs. The distribution of length of the longest positive LCRs, second-longest positive LCRs, and third-longest positive LCRs in each protein in each data set are presented in Fig. 5 *A*. The longest positive LCRs in FG Nups (labeled as FG-1st in Fig. 5 *A*) feature a distinct distribution compared with the second- and third-longest positive LCRs, as well as all the longest, second-longest, and third-longest positive LCRs in DisProt. The longest positive LCRs in FG Nups are longer than in the other five categories. As an additional analysis, we measured the gap between the length of the longest and second-longest positive LCR as well as the gap between the length of the second-longest positive LCR and third-longest positive LCR in each protein in FG Nups and DisProt. Our goal was to see whether the length gap distribution of the longest and second-longest positive LCRs in FG Nups is different from the other three categories. Results in Fig. 5 *B* show that the gap between the longest and second-longest positive LCR in FG Nups features a distinct distribution compared with the other three categories. The same analysis was done only for the ratio of the longest LCR/second-longest LCR and second-longest LCR/third-longest LCR. Similar to the previous analysis, the ratio between the longest positive LCR and the second-longest positive LCR in FG Nups is larger than the three other categories (Fig. 5 *C*). The results in Fig. 5 show how distinct the lpLCRs in FG Nups are from the rest of the LCRs in both FG Nups and DisProt. Our data suggest that lpLCRs in FG Nups are generated by a distinct process that makes them much longer than randomly generated LCRs in FG Nups and the other data sets.

For a more marked representation of lpLCRs in FG Nups, we excluded LCRs shorter than 20 and 40 amino acids, respectively (Fig. S3, *b and c*). Because shorter LCRs are excluded, expectedly, average length is increased for all four data groups. However, the average for positive LCRs in FG Nups reaches significantly higher values compared with the other three categories (Fig. S3, *b and c*). Considering that short LCRs are formed randomly because of the high density of charged residues in some regions, removing them from the data provides a better insight about the length of nonrandom LCRs. Based on these results, the average length of positive LCRs in FG Nups is two- to threefold higher than that of negative LCRs in FG Nups, as well as positive and negative LCRs in DisProt.

To further explore the differences in charge distribution of the two data sets, we examined the fraction of each amino acid sequence that is covered by LCRs. Lengths of all of the LCRs present in each sequence were added and then normalized by the length of the sequence. We call this factor "LCR-covered percentage" (Fig. 6). This metric was evaluated for proteins in both data sets for several different minimal LCR lengths. As the LCR threshold increases from 2 to 20 and then 40, all of the histograms shift strongly toward the left, meaning that it is rare for a sequence to be covered by long LCR regions. In general, when short LCRs are excluded, LCRs make up a very small percentage of DisProt sequences, and negative LCRs make up a small percentage of FG Nups. However, positive LCRs in FG Nups do not demonstrate the same behavior. When considering the LCRs of larger than 40 amino acids, DisProt sequences will have a low positive and negative LCR-covered

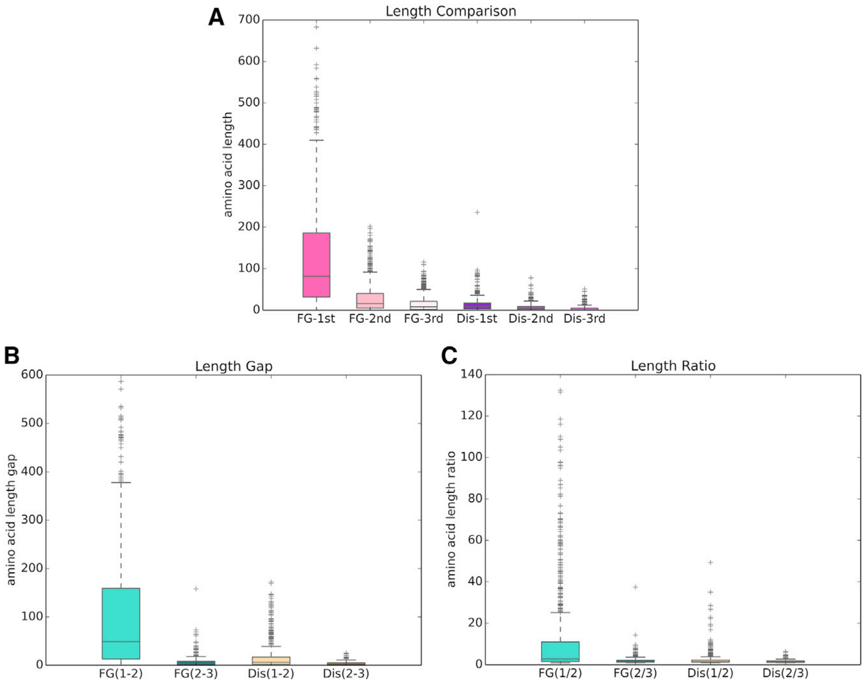

FIGURE 5 Comparison between the length of the lpLCRs and other LCRs in FG Nups and DisProt. (*A*) Boxplot of length of longest positive LCRs, second-longest positive LCRs, and third-longest positive LCRs in each protein in FG Nups and DisProt data sets (briefly mentioned as FG and Dis, respectively). Longest positive LCRs in FG Nups are significantly longer than second- and third-longest positive LCRs in FG Nups and all of the LCRs in DisProt. (*B*) Boxplot of the gap between length of first- and second-longest positive LCRs (shown as FG (1,2) and Dis (1,2)) and second- and third-longest positive LCRs (shown as FG (2,3) and Dis (2,3)) for proteins in FG Nups and DisProt (briefly mentioned as FG and Dis, respectively). The gap between the length of the longest and second-longest positive LCR in FG Nups is much larger than the gap between the second- and third-longest positive LCRs in FG Nups and the gap between the longest and second-longest and the gap between second-longest and third-longest positive LCRs in DisProt. (*C*) Boxplot of the ratio of the length of first- and second-longest positive LCRs (shown as FG(1/2) and Dis(1/2)) and second- and third-longest positive LCRs (shown as FG(2/3) and Dis(2/3)) for proteins in FG Nups and DisProt (briefly mentioned as FG and Dis, respectively). The ratio between the length of first and second positive LCRs in FG Nups is higher than the other three categories. All three panels show that the distribution of length of longest positive LCRs in FG Nups is different from the other categories, and lpLCRs in FG Nups are significantly longer than other LCRs. To see this figure in color, go online.

percentage and FG Nups will have a low negative LCR-covered percentage, but FG Nups will still have a high positive LCR-covered percentage. This implies that the high LCR-covered percentage for FG Nup positive LCRs is due to presence of lpLCRs, but for the other categories, it is due to presence of several short LCRs.

DISCUSSION

We previously identified lpLCRs as an evolutionarily conserved feature in FG Nup sequences (12). In this study, we further demonstrate that these features are virtually exclusive to FG Nups and are not found in other disordered proteins. Although FG Nups have the same density of hydrophobic amino acids as the average DisProt protein (Fig. 2), they are differentiated by their low charged residue content and their distinctive charged residue distributions. Based on the analysis presented in Fig. 3 and Fig. 4, these differences are explained by the presence of exceedingly long lpLCRs in FG Nups. Our data suggest that these lpLCRs are virtually exclusive to FG Nups, even in a data set of half a million proteins.

Charged amino acids are disorder promoting and common in disordered proteins such as those in DisProt (42). Because FG Nups fall under the same category of disordered proteins, they typically have similar properties. Therefore, they contain subsequences that are charge rich and look similar to DisProt sequences. However, our work identifies specific regions (lpLCRs) in FG Nups that behave entirely differently and are responsible for many of the differences between FG Nups and disordered proteins overall. As presented in Fig. 4, removing lpLCRs from the FG Nups data set to leave only the normal regions makes it similar to DisProt in terms of charge distribution. General DisProt proteins and the charge-rich regions of FG Nups both have a high charge density, and their charged residues form many small LCRs that cover a large portion of the sequence (Fig. 6). However, the lpLCRs in FG Nups have very low charge densities (please see Fig. 3) and generally obey different statistics. The low charge density of lpLCRs is the factor lowering the charge density of FG Nups below that of other disordered proteins.

Data from this study and our previous work (12) show that the lpLCRs in FG Nups have substantially different properties from regular proteins, indicating that the origin and function of these small and large LCRs are different. Small LCRs found in all disordered proteins can be considered to be mainly randomly formed, whereas the lpLCRs are embedded in the sequence to facilitate a function. This is demonstrated by the actual behavior of the proteins, in which charge-rich segments form a rather straight conformation (6,17), whereas the charge-poor regions (lpLCRs) (12) form more compact conformations (13). The consistent occurrence of lpLCRs in FG Nups, despite their almost complete absence in other proteins, indicates that they are evolutionarily conserved and likely correlated with the

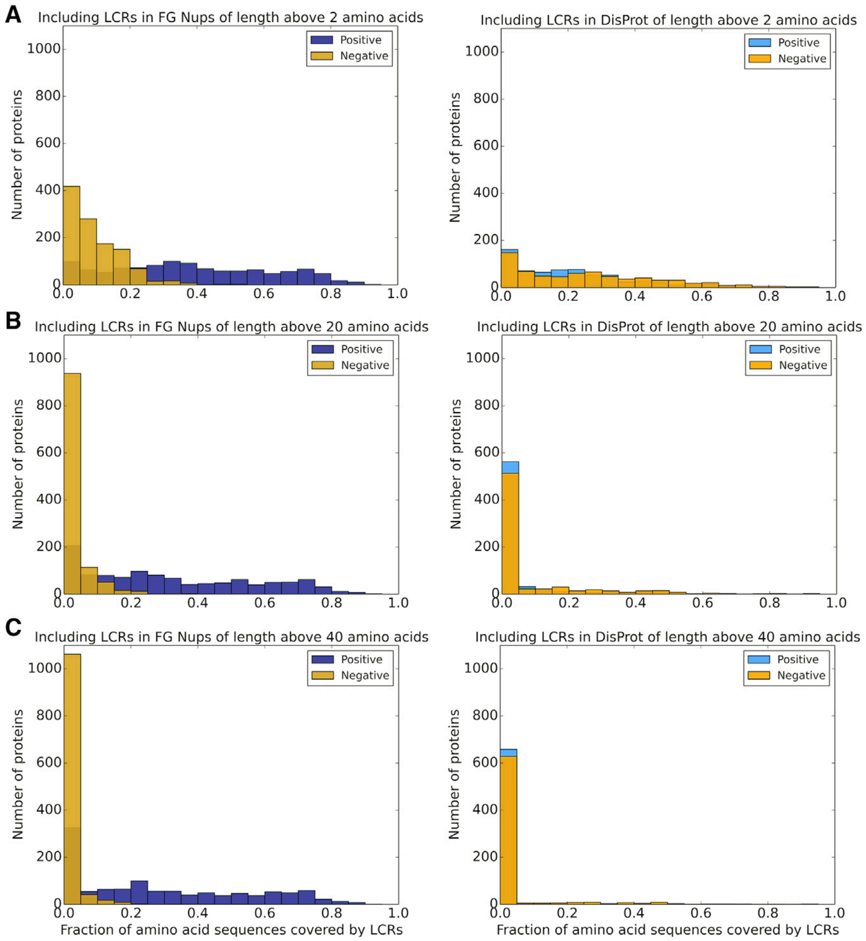

FIGURE 6 Histogram of positive and negative LCR-covered percentage in FG Nups and DisProt data sets. By increasing the threshold of the length of the LCRs considered in the data (from 2 (*A*) to 20 (*B*) to 40 (*C*)), the LCR-covered percentage decreases for negative LCRs in FG Nups and positive and negative LCRs in DisProt but will still hold considerably large values for positive LCRs in FG Nups. This indicates that the high positive LCR-covered percentage in FG Nups is caused by lpLCRs, whereas the large values of the LCR-covered percentage for the other three categories were the result of the presence of several short LCRs. To see this figure in color, go online.

specific function of FG Nups in nucleocytoplasmic transport (13).

The results of this study suggest that two domains can be observed in the sequences of FG Nups considering charge distribution. One domain is rich in charged amino acids and therefore forms a relaxed-coil conformation. This domain includes several negative and positive short-length LCRs that are randomly formed because of the high density of charged residues. This domain is usually the only domain that exists in DisProt proteins (in terms of charge distribution). The other domains existing in FG Nups sequences are extended subsequences that contain only positively charged residues and a low charge density. This is a unique sequence feature that shows up only once along the amino acid sequences of FG Nups. These patterns (named lpLCRs) are evolutionarily conserved, virtually exclusive to FG Nups, and what differentiate FG Nups from DisProt in terms of charge distribution. We had also previously shown that these patterns are mostly located toward the N-terminus of FG Nups, which is toward the center of the NPC (12), and make the permeability barrier at the center of the NPC. All of the abovementioned facts about lpLCRs strongly imply that they are important for the conformation and function of FG Nups.

Our previous coarse-grained molecular dynamics study on the effect of lpLCRs suggests that mutation of charged residues to Alanine in the lpLCR makes the conformation of FG Nups collapse to form a highly aggregated conformation at the center of the NPC (13). Furthermore, our recent simulations (44) suggested a regulatory role for lpLCRs in FG Nup conformation and interactions with cargo complexes. Our simulations implied that lpLCRs exist because the function of FG Nups relies on a charge density low enough for them to form an aggregated conformation (permeability barrier) but high enough to prevent “sticky” interactions with cargo complexes. As a result, the few charges in the lpLCR prevent the FG Nups from forming highly aggregated conformations while allowing for a dense permeability barrier to form at the center of the NPC (45). Additionally, the presence of positive charges at the NPC’s permeability barrier is desirable and a key feature for transport of negatively charged transport receptors (43).

It is important to note that lpLCRs are features that are not observed in every FG Nup, but most of them. Some FG Nups do not feature this pattern because their function relies on forming relaxed-coil conformations. As an example, in the yeast NPC, Nup159, Nup60, and Nup1 do not feature lpLCRs. Interestingly, these Nups are located at the

extremities of the yeast NPC. Our unpublished results show that because these FG Nups are charge rich and their amino acid sequences lack the presence of lpLCRs, these FG Nups do not form aggregated conformations and leave the extremities of the NPC open. On the other hand, the rest of the FG Nups in yeast form aggregated conformations inside the NPC. Short Nups such as Nup49 and Nup57 aggregate toward the scaffold of the NPC, whereas the other FG Nups form an aggregated conformation at the center of the NPC, reminiscent of the permeability barrier phenomenon [13],[45]). Therefore, the absence of large positive LCRs in some FG Nups may be correlated with their location and function.

CONCLUSIONS

This study shows that FG Nups are different from other disordered proteins not only in terms of fraction of charged residues but also in terms of charge distribution and charge decoration. The difference is primarily driven by the presence of large subsequences in FG Nups that contain only positively charged residues, have a low charge density, and are located toward the N-terminus of these proteins. These patterns, named lpLCRs, are evolutionarily conserved, occur once in the sequences of FG Nups, and are virtually exclusive to FG Nups. On removal of these subsequences from the data set, FG Nups become similar to DisProt proteins in terms of charge distribution. These findings imply that lpLCRs are essential for the function of FG Nups. Our previously published data, as well as our ongoing research, reveal that the presence of lpLCRs significantly affects the conformation of FG Nups and their interaction with cargo complexes.

SUPPORTING MATERIAL

Supporting material can be found online at https://doi.org/10.1016/j.bpj.2021.06.031.

AUTHOR CONTRIBUTIONS

M.P., M.S., and M.R.K.M. designed the experiments. M.P., M.S., V.S.N., and A.M.D. performed the experiments. M.P., M.S., V.S.N., A.M.D., and M.R.K.M. analyzed the data and wrote the manuscript.

ACKNOWLEDGMENTS

The authors acknowledge members of the molecular cell biomechanics laboratory for their feedback and fruitful discussions.

Financial support by the National Science Foundation through grant CMMI-1538707 is gratefully acknowledged.

REFERENCES

1. Hampoelz, B., A. Andres-Pons, …, M. Beck. 2019. Structure and assembly of the nuclear pore complex. *Annu. Rev. Biophys.* 48:515–536.
2. Beck, M., and E. Hurt. 2017. The nuclear pore complex: understanding its function through structural insight. *Nat. Rev. Mol. Cell Biol.* 18:73–89.
3. Jovanovic-Talisman, T., and A. Zilman. 2017. Protein transport by the nuclear pore complex: simple biophysics of a complex biomachine. *Biophys. J.* 113:6–14.
4. Raveh, B., J. M. Karp, …, D. Cowburn. 2016. Slide-and-exchange mechanism for rapid and selective transport through the nuclear pore complex. *Proc. Natl. Acad. Sci. USA.* 113:E2489–E2497.
5. Li, C., A. Goryaynov, and W. Yang. 2016. The selective permeability barrier in the nuclear pore complex. *Nucleus.* 7:430–446.
6. Yamada, J., J. L. Phillips, …, M. F. Rexach. 2010. A bimodal distribution of two distinct categories of intrinsically disordered structures with separate functions in FG nucleoporins. *Mol. Cell. Proteomics.* 9:2205–2224.
7. Huang, K., M. Tagliazucchi, …, I. Szleifer. 2019. Molecular model of the nuclear pore complex reveals a thermoreversible FG-network with distinct territories occupied by different FG motifs. *bioRxiv* https://doi.org/10.1101/568865.
8. Ando, D., M. Colvin, …, A. Gopinathan. 2013. Physical motif clustering within intrinsically disordered nucleoporin sequences reveals universal functional features. *PLoS One.* 8:e73831.
9. Ando, D., R. Zandi, …, A. Gopinathan. 2014. Nuclear pore complex protein sequences determine overall copolymer brush structure and function. *Biophys. J.* 106:1997–2007.
10. Ghavami, A., L. M. Veenhoff, …, P. R. Onck. 2014. Probing the disordered domain of the nuclear pore complex through coarse-grained molecular dynamics simulations. *Biophys. J.* 107:1393–1402.
11. Tagliazucchi, M., O. Peleg, …, I. Szleifer. 2013. Effect of charge, hydrophobicity, and sequence of nucleoporins on the translocation of model particles through the nuclear pore complex. *Proc. Natl. Acad. Sci. USA.* 110:3363–3368.
12. Peyro, M., M. Soheilypour, …, M. R. K. Mofrad. 2015. Evolutionarily conserved sequence features regulate the formation of the FG network at the center of the nuclear pore complex. *Sci. Rep.* 5:15795.
13. Peyro, M., M. Soheilypour, …, M. R. K. Mofrad. 2015. Nucleoporin's like charge regions are major regulators of FG coverage and dynamics inside the nuclear pore complex. *PLoS One.* 10:e0143745.
14. Müller-Späth, S., A. Soranno, …, B. Schuler. 2010. From the cover: charge interactions can dominate the dimensions of intrinsically disordered proteins. *Proc. Natl. Acad. Sci. USA.* 107:14609–14614.
15. Mao, A. H., S. L. Crick, …, R. V. Pappu. 2010. Net charge per residue modulates conformational ensembles of intrinsically disordered proteins. *Proc. Natl. Acad. Sci. USA.* 107:8183–8188.
16. Hofmann, H., A. Soranno, …, B. Schuler. 2012. Polymer scaling laws of unfolded and intrinsically disordered proteins quantified with single-molecule spectroscopy. *Proc. Natl. Acad. Sci. USA.* 109:16155–16160.
17. Das, R. K., and R. V. Pappu. 2013. Conformations of intrinsically disordered proteins are influenced by linear sequence distributions of oppositely charged residues. *Proc. Natl. Acad. Sci. USA.* 110:13392–13397.
18. Das, R. K., K. M. Ruff, and R. V. Pappu. 2015. Relating sequence encoded information to form and function of intrinsically disordered proteins. *Curr. Opin. Struct. Biol.* 32:102–112.
19. Schuler, B., A. Soranno, …, D. Nettels. 2016. Single-molecule FRET spectroscopy and the polymer physics of unfolded and intrinsically disordered proteins. *Annu. Rev. Biophys.* 45:207–231.
20. Huihui, J., T. Firman, and K. Ghosh. 2018. Modulating charge patterning and ionic strength as a strategy to induce conformational changes in intrinsically disordered proteins. *J. Chem. Phys.* 149:085101.
21. Beveridge, R., L. G. Migas, …, P. E. Barran. 2019. Ion mobility mass spectrometry uncovers the impact of the patterning of oppositely charged residues on the conformational distributions of intrinsically disordered proteins. *J. Am. Chem. Soc.* 141:4908–4918.
22. Martin, E. W., A. S. Holehouse, …, T. Mittag. 2016. Sequence determinants of the conformational properties of an intrinsically disordered

protein prior to and upon multisite phosphorylation. *J. Am. Chem. Soc.* 138:15323–15335.

23. Best, R. B. 2017. Computational and theoretical advances in studies of intrinsically disordered proteins. *Curr. Opin. Struct. Biol.* 42:147–154.
24. Uversky, V. N., and A. K. Dunker. 2010. Understanding protein non-folding. *Biochim. Biophys. Acta.* 1804:1231–1264.
25. Dunker, A. K., J. D. Lawson, ..., Z. Obradovic. 2001. Intrinsically disordered protein. *J. Mol. Graph. Model.* 19:26–59.
26. Uversky, V. N., J. R. Gillespie, and A. L. Fink. 2000. Why are "natively unfolded" proteins unstructured under physiologic conditions? *Proteins.* 41:415–427.
27. Uversky, V. N. 2002. Natively unfolded proteins: a point where biology waits for physics. *Protein Sci.* 11:739–756.
28. Zarin, T., B. Strome, ..., A. M. Moses. 2019. Proteome-wide signatures of function in highly diverged intrinsically disordered regions. *eLife.* 8:e46883.
29. Zarin, T., C. N. Tsai, ..., A. M. Moses. 2017. Selection maintains signaling function of a highly diverged intrinsically disordered region. *Proc. Natl. Acad. Sci. USA.* 114:E1450–E1459.
30. Daughdrill, G. W., P. Narayanaswami, ..., C. J. Brown. 2007. Dynamic behavior of an intrinsically unstructured linker domain is conserved in the face of negligible amino acid sequence conservation. *J. Mol. Evol.* 65:277–288.
31. Uversky, V. N. 2016. Dancing protein clouds: the strange biology and chaotic physics of intrinsically disordered proteins. *J. Biol. Chem.* 291:6681–6688.
32. Uversky, V. N. 2019. Intrinsically disordered proteins and their "mysterious" (Meta)Physics. *Front. Phys.* 7:10.
33. Srivastava, D., and M. Muthukumar. 1996. Sequence dependence of conformations of polyampholytes. *Macromolecules.* 29:2324–2326.
34. Sawle, L., and K. Ghosh. 2015. A theoretical method to compute sequence dependent configurational properties in charged polymers and proteins. *J. Chem. Phys.* 143:085101.
35. Firman, T., and K. Ghosh. 2018. Sequence charge decoration dictates coil-globule transition in intrinsically disordered proteins. *J. Chem. Phys.* 148:123305.
36. Beh, L. Y., L. J. Colwell, and N. J. Francis. 2012. A core subunit of Polycomb repressive complex 1 is broadly conserved in function but not primary sequence. *Proc. Natl. Acad. Sci. USA.* 109:E1063–E1071.
37. Moussavi-Baygi, R., and M. R. K. Mofrad. 2016. Rapid Brownian motion primes ultrafast reconstruction of intrinsically disordered Phe-Gly repeats inside the nuclear pore complex. *Sci. Rep.* 6:29991.
38. Walsh, I., A. J. M. Martin, ..., S. C. E. Tosatto. 2012. ESpritz: accurate and fast prediction of protein disorder. *Bioinformatics.* 28:503–509, Published online December 20, 2011.
39. Piovesan, D., F. Tabaro, ..., S. C. E. Tosatto. 2017. DisProt 7.0: a major update of the database of disordered proteins. *Nucleic Acids Res.* 45:D219–D227.
40. Hatos, A., B. Hajdu-Soltész, ..., D. Piovesan. 2020. DisProt: intrinsic protein disorder annotation in 2020. *Nucleic Acids Res.* 48:D269–D276.
41. Oates, M. E., P. Romero, ..., J. Gough. 2013. D^2P^2: database of disordered protein predictions. *Nucleic Acids Res.* 41:D508–D516.
42. Campen, A., R. M. Williams, ..., A. K. Dunker. 2008. TOP-IDP-scale: a new amino acid scale measuring propensity for intrinsic disorder. *Protein Pept. Lett.* 15:956–963.
43. Colwell, L. J., M. P. Brenner, and K. Ribbeck. 2010. Charge as a selection criterion for translocation through the nuclear pore complex. *PLoS Comput. Biol.* 6:e1000747.
44. Peyro, M., A. M. Dickson, and M. R. K. Mofrad. 2021. Nucleoporins' exclusive amino acid sequence features regulate their transient interaction with and selectivity of cargo complexes in the nuclear pore. *Molecular Biology of the Cell*, In press.
45. Matsuda, A., and M. R. K. Mofrad. 2021. Free Energy Calculations Shed Light on the Nuclear Pore Complex's Selective Barrier Nature. *Biophysical Journal*, In press. https://doi.org/10.1016/j.bpj.2021.07.025.

Biophysical Journal, Volume *120*

Supplemental information

FG nucleoporins feature unique patterns that distinguish them from other IDPs

Mohaddeseh Peyro, Mohammad Soheilypour, Vikrum S. Nibber, Andrew M. Dickson, and Mohammad R.K. Mofrad

SUPPLEMENTARY MATERIALS

FG Nucleoporins feature unique amino acid sequence patterns that distinguish them from other IDPs

Mohaddeseh Peyro[1,§], Mohammad Soheilypour[1,§], Vikrum S. Nibber[1], Andrew M. Dickson[1], Mohammad R.K. Mofrad[1,2,*]

[1] Molecular Cell Biomechanics Laboratory, Departments of Bioengineering and Mechanical Engineering, University of California, Berkeley, Berkeley, CA 94720
[2] Molecular Biophysics and Integrative Bioimaging Division, Lawrence Berkeley National Laboratory, Berkeley, CA 94720

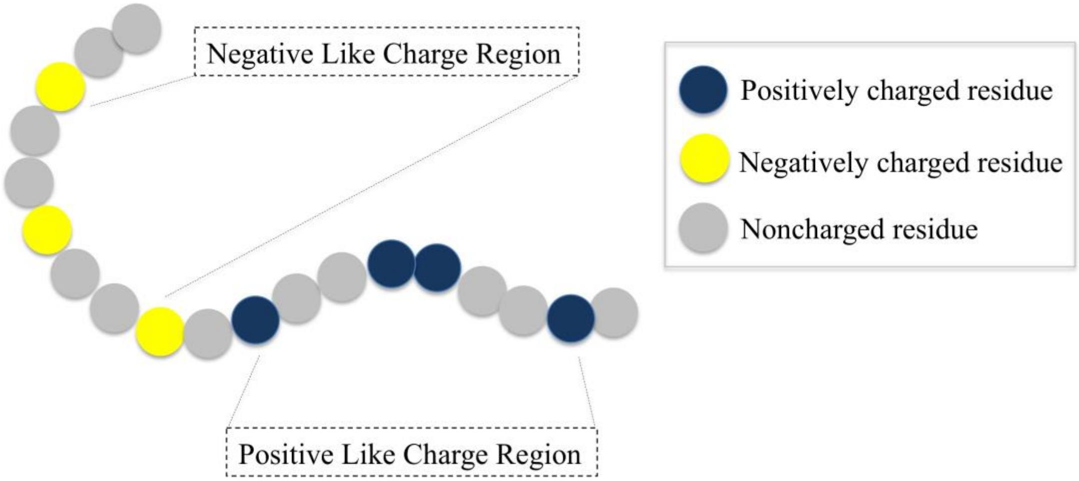

Fig. S 1: Hypothetical disordeded region of a protein containing a positive like charge region (LCR), with a length of 8 and charge count of 4, followed by a negative LCR, with a length of 7 and charge count of 3.

[§]These authors contributed equally to this work.
[*]Corresponding author: mofrad@berkeley.edu

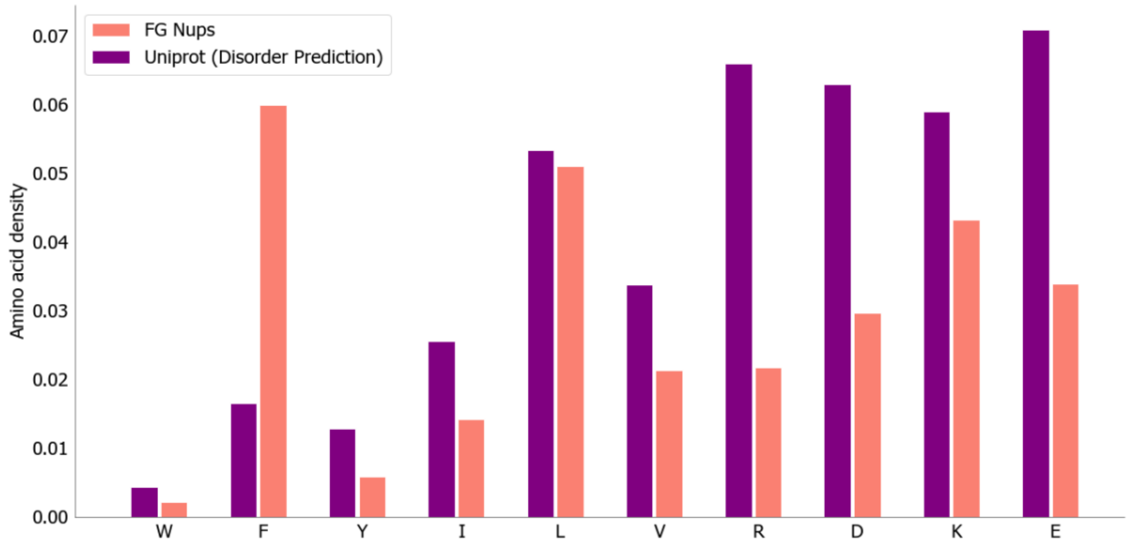

Fig. S 2: Comparison of abundance of the most order-promoting and the most disorder-promoting amino acids in FG Nups vs. UniProt (disorder prediction). FG Nups have a lower abundance of all of the order-promoting amino acids except for F. The sum of the abundance of order-promoting amino acids are nearly equal. On the other hand, the abundance of all of the charged residues is lower in FG Nups compared to UniProt (disorder prediction)

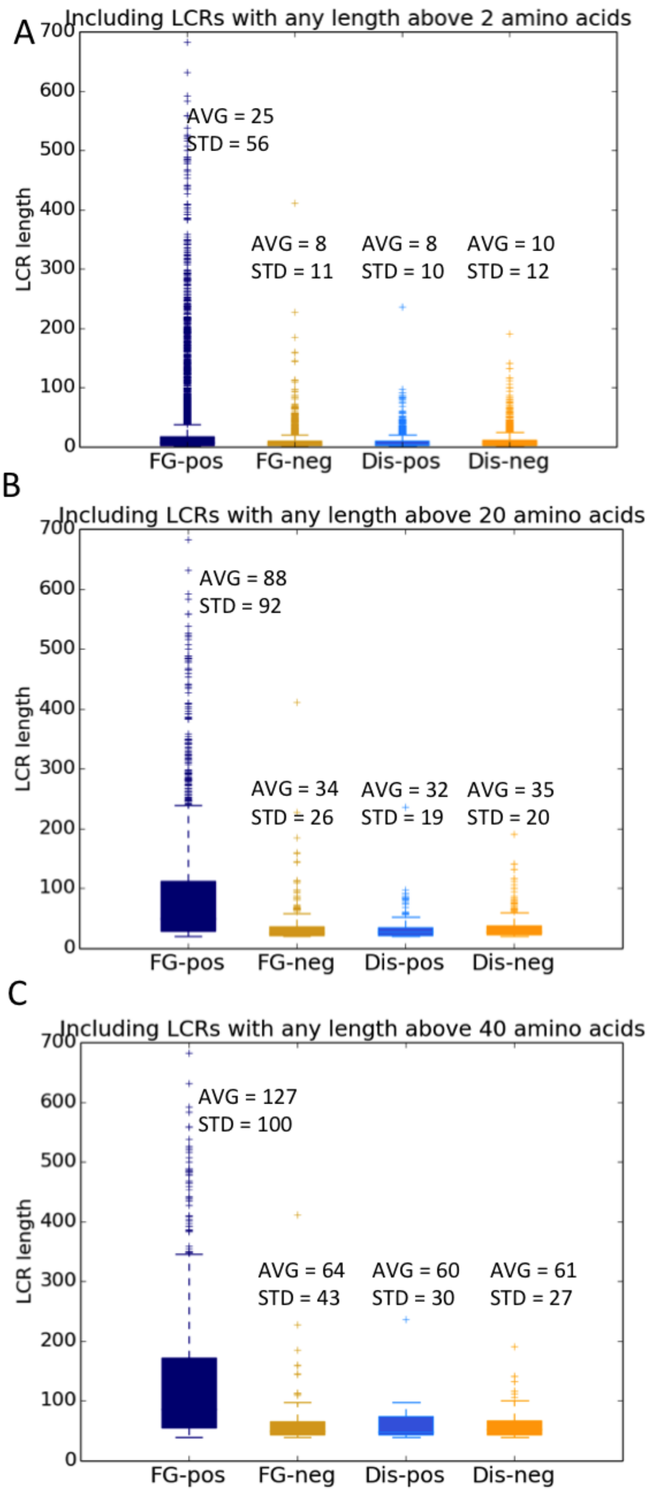

Fig. S 3: Boxplots of length of positive LCRs in FG Nups, negative LCRs in FG Nups, positive LCRs in DisProt and negative LCRs in DisProt A) Considering LCRs of any length (above 2 amino acids), the average length of positive LCRs in FG Nups is 3 times higher than the other three categories. Panels (B) and (C)) Removing shorter LCRs (below 20 in panel B and below 40 in panel C) increases the average length of all the categories, but average length of positive LCRs in FG Nups remains significantly higher than the other three categories, and emphasizes the difference in the length distribution of positive LCRs in FG Nups.
